# The Timecourses of Functional, Morphological, and Molecular Changes Triggered by Light Exposure in Sprague–Dawley Rat Retinas

**DOI:** 10.3390/cells10061561

**Published:** 2021-06-21

**Authors:** Serena Riccitelli, Mattia Di Paolo, James Ashley, Silvia Bisti, Stefano Di Marco

**Affiliations:** 1 Department of Biotechnological and Applied Clinical Sciences, University of L’Aquila, 67100 L’Aquila, Italy; serena.riccitelli@weizmann.ac.il (S.R.); m.dipaolo@bio-aurum.it (M.D.P.); Silvia.Bisti@iit.it (S.B.); 2 School of Biological Sciences, The University of Manchester, Manchester M13 9PL, UK; jamash22@gmail.com; 3 Istituto Nazionale di Biostrutture e Biosistemi (INBB), 00136 Roma, Italy; 4 Center for Synaptic Neuroscience and Technology, Istituto Italiano di Tecnologia, 16132 Genova, Italy; 5 IRCCS, Ospedale Policlinico San Martino, 16132 Genova, Italy

**Keywords:** light damage, neurodegeneration, functional analysis, early detection, remodeling

## Abstract

Retinal neurodegeneration can impair visual perception at different levels, involving not only photoreceptors, which are the most metabolically active cells, but also the inner retina. Compensatory mechanisms may hide the first signs of these impairments and reduce the likelihood of receiving timely treatments. Therefore, it is essential to characterize the early critical steps in the neurodegenerative progression to design adequate therapies. This paper describes and correlates early morphological and biochemical changes in the degenerating retina with in vivo functional analysis of retinal activity and investigates the progression of neurodegenerative stages for up to 7 months. For these purposes, Sprague–Dawley rats were exposed to 1000 lux light either for different durations (12 h to 24 h) and examined seven days afterward (7d) or for a fixed duration (24 h) and monitored at various time points following the exposure (up to 210d). Flash electroretinogram (fERG) recordings were correlated with morphological and histological analyses to evaluate outer and inner retinal disruptions, gliosis, trophic factor release, and microglial activation. Twelve hours or fifteen hours of exposure to constant light led to a severe retinal dysfunction with only minor morphological changes. Therefore, early pathological signs might be hidden by compensatory mechanisms that silence retinal dysfunction, accounting for the discrepancy between photoreceptor loss and retinal functional output. The long-term analysis showed a transient functional recovery, maximum at 45 days, despite a progressive loss of photoreceptors and coincident increases in glial fibrillary acidic protein (GFAP) and basic fibroblast growth factor-2 (bFGF-2) expression. Interestingly, the progression of the disease presented different patterns in the dorsal and ventral retina. The information acquired gives us the potential to develop a specific diagnostic tool to monitor the disease’s progression and treatment efficacy.

## 1. Introduction

The quality of vision dramatically impacts the quality of human life [1]. Many ocular pathologies induced by several factors, such as gene mutations, environmental stresses, metabolic dysfunction, and aging, lead to reduced visual performance and eventually complete blindness caused by inflammation, mitochondrial dysfunction, synaptic remodeling, and neuronal death [2]. These pathologies include a heterogeneous group of photoreceptor degenerations whereby populations of rods and cones are distinctly affected, i.e., rods are primarily involved in retinitis pigmentosa (RP) [3], cones in age-related macular degenerations (AMD) [3], and both rods and cones simultaneously in Leber congenital amaurosis (LCA) [4] and Stargardt’s disease (STGD1, autosomal recessive [5]). Although a variety of new therapeutic strategies have been suggested (including stem cell transplantation to replace photoreceptors [6,7], bionic implants [8,9], and optogenetics approaches [10,11,12]), currently there are no effective cures. Common pharmacological approaches studied on rodents [13,14] and tested on humans [15,16,17,18] have as their primary objective slowing down photoreceptor death to preserve surviving retinal function, increase tissue resilience, and prevent retinal reorganization. Indeed, it is well known that photoreceptors stress initiate an unavoidable chain of events, collectively termed retinal remodeling [19,20], in the remnant inner retina, independently of the molecular defects that initially trigger retinal degeneration [21,22,23,24]. Abnormal reprogramming occurs in all pathology phases and can begin even before photoreceptor death [22,25]. In their final states, these modifications could lead to considerable changes in the receptive field properties of retinal ganglion cells, the retina’s output cells, impinging consequently on the transmission of visual information to the brain [26,27,28].

Retinal degeneration has at least three related and recognized phases [29]. The first is the irreversible death of photoreceptors—cells with high metabolic activity predisposed to a wide range of insults, including high-intensity light [3]. The second is the dysfunction of surviving photoreceptors and their subsequent loss [30,31,32]. The third is related to profound structural and functional abnormalities in the inner retina (i.e., neuronal cell death, microglia migration, rewiring of retinal circuits, and glial hypertrophy) [33].

With these factors in mind, there is a need to diagnose visual impairments at early stages to increase the chances of an effective pharmacological treatment. From a therapeutic perspective, it is also essential to define the critical steps during neurodegenerative progression to design adequate therapies that arrest remodeling events or target specific cell subtypes in the remnant tissue.

To address these clinical needs, we planned an experimental protocol with two primary aims:

1. To characterize and correlate early morphological and biochemical changes occurring in the degenerating retina with in vivo functional analysis of retinal activity.
2. To investigate the progression of neurodegenerative stages.

In the current work, we used a consolidated rat model of light-induced retinal degeneration, previously shown to mimic some aspects of AMD (the presence of oxidative stress [34], inflammatory processes [35], and photoreceptor death [36]). Indeed, apart from the relevance of this model in AMD research, we exploited its well-known characteristics to follow a neurodegenerative process that starts in the dorsal retina and spreads over time. Specifically, by using two different protocols (<24 h and 24 h of light exposure) and a combination of histological and electrophysiological investigations, we first determined the minimal duration of bright light exposure (1000 lux, white light) necessary to induce detectable retinal functional alterations. Interestingly, our findings revealed that functional changes precede anatomical ones. In the second stage, we investigated the temporal course of the neurodegeneration following 24 h of light exposure while extending our analyses to the inner retina by relying on antibodies against cell-type-specific markers and trophic factors.

Altogether, the knowledges acquired will allow us to extrapolate information (i.e., emergent ERG signals) that might help to detect the pathology through functional tests before a large portion of photoreceptors is already injured, the retina severely damaged, and neuronal circuitry remodeled. Secondly, following alterations over time will provide valuable insights for dissecting general retinal degeneration pathways that might help develop treatments based on different dystrophy stages.

## 2. Materials and Methods

### 2.1. Animals

According to the ARVO Statement for the “Use of Animals in Ophthalmic and Vision Research”, all experiments were carried out with authorization numbers 448/2016-PR and 862/2018-PR, issued by the Ministry of Health, and approved by the local Ethical Committee of the University of L’Aquila.

### 2.2. Light Exposure Protocols

Albino Sprague–Dawley (SD) rats were born and housed at the University of L’Aquila animal facilities in dim cyclic light conditions (12 h light, 12 h dark), at an ambient light level of approximately 5 lux, with food and water available ad libitum.

Data reported in the present study were obtained from experiments conducted on healthy control (HC) animals and animals treated with intense white light, commonly referred to as “light damaged” (LD), which were divided into twelve groups, as depicted in Table 1. Light damage was generated by placing adult albino rats into singular plexiglass cages with lights positioned at the top and at the bottom to ensure an iso-luminance environment (1000 lux, monitored through a lux meter) inside the cage. The litter was removed from the cage to prevent rats from hiding their eyes from the light. Light exposure started at the beginning of the day, immediately after 12 h of darkness. We analyzed different combinations of exposure durations and periods of time following the end of light exposure in normal light conditions. Accordingly, animals were exposed to 1000 lux light for different durations (12 h, 15 h, 18 h, and 24 h) and allowed to recover for a post-exposure period of 7 days (7d) in dim cyclic illumination conditions in standard cages (5 lux, cyclic light). The second group of animals was exposed to the same light intensity (1000 lux) for 24 h and sacrificed at different recovery periods (after 0, 15, 30, 45, 60, 90, and 210 days (d)). The same healthy control (HC) group, raised at 5 lux in cyclic light, and the group exposed to light for 24 h and left to recover for 7 days (LD24h7d), were used in the two experiments. In the text, we refer to short-term exposure to indicate 12 h of light exposure.

### 2.3. Electrophysiological Recordings and Data Analysis

We performed flash electroretinogram (fERG) recordings in controls and evaluated retinal function in each experimental group following the light exposure treatment. To minimize variability among groups, electrophysiological recordings were performed before exposing animals to LD (a pull of these recordings were used as HC, assuming no age-related changes in our temporal window of interest [37,38]). Albino rats were previously dark-adapted overnight, and electroretinograms were recorded in a dark room [14]. Briefly, animals were anesthetized with an intraperitoneal injection of ketamine/xylazine (keta-vet 100 mg/mL, Intervet production Srl, and xylazine 1 g, Sigma Co., at 10 and 1.2 mg/100 g, respectively). Corneas were anesthetized with a drop of Novocaine, and pupils were dilated with 1.0% mydriacyl tropicamide. Animals were mounted on a stereotaxic apparatus and positioned inside the Ganzfeld dome’s opening (Biomedica Mangoni, Pisa, Italy). Body temperature was maintained at 37.5 °C using a heating pad controlled by a rectal temperature probe. Recordings were carried out for both eyes simultaneously, with a platinum electrode loop being placed on each cornea, and individually considered. The reference electrodes were inserted subcutaneously in the proximity of the eyes, and the ground electrode was inserted in the anterior scalp, between the eyes. The standard ERG protocol advocated by the ISCEV (International Society for Clinical Electrophysiology of Vision) was used [39]. Responses were recorded at progressively brighter short flashes (0.001–100 cd*s/m^2^ range, from scotopic to photopic) over 450 ms, plus 50 ms of pre-trial baseline. Responses were amplified differentially, bandpass filtered at 0.3–300 Hz, and sampled at 16.3 kHz. To reduce variability and background noise, responses were averaged (n = 3 per each luminance), with an inter-stimulus interval (ISI) ranging from 60 s for lower intensities to 5 min for the three brightest flashes. We evaluated the amplitudes (in μV) of the a-wave (baseline to the first negative deflection), b-wave (from a-wave peak to positive b-wave peak), and oscillatory potentials (the high-frequency wavelengths residing on the leading edge of the b-wave separated from the ERG using a high-pass filter. The amplitudes of wavelets OP1–4, measured from the trough to the peak of each response component, were summed) (see Figure 5a,f). For photopic recordings (light-adapted ERG, cone response), rats were light-adapted to background illumination (30 cd*s/m^2^, 10 min), and 20 replicate responses elicited by white light (100 cd*s/m^2^) at a pulse frequency of 1 Hz were averaged. A 30 Hz photopic flicker response was also obtained using the same background illumination and stimulus intensity. One hundred replicate responses were averaged. The first and the second harmonic magnitudes were determined by performing FFT analysis with a compiled routine (see Figure 6a,c).

In some cases, measurements were obtained from the same animal at successive survival times following light exposure cessation (not more than three ERG sessions, including the initial one, were performed in total for a single animal). When the amplitude of either an a-wave or a b-wave was not higher than at least 1 SD (not measurable) across the entire recording session, the retina was discarded from the subsequent analyses (more common in the later stages of the degeneration, i.e., LD24h210d). Custom-written procedures in IGOR Pro 6.3 (Wavemetrics, Lake Oswego, OR, USA) software were used to analyze electrophysiological data. The distributions of ERG response across several experimental groups at different light intensities were described by means and standard errors. Two-way ANOVA followed by the Tukey post hoc test was used to compare the ERG parameters (a, b, and OP amplitudes) between groups of animals for each flash intensity used. One-way ANOVA followed by the Tukey post hoc test was used to analyze statistical significance in the photopic recording (photopic ERG and flicker ERG, a single flash intensity used).

### 2.4. Tissue Processing, Morphological Analyses, and Immunostaining Protocols

At the end of the last recording session, rats were sacrificed by CO_2_ inhalation. Both eyes were enucleated and fixed in 4% paraformaldehyde for 6 h at 4°C and washed in 0.1 M phosphate-buffered saline (PBS, pH 7.4). The eyes were further processed to obtain an “eyecup” consisting of the sclera, choroid, and retina. Eyecups were cryoprotected by immersion in 10%, 20%, and 30% sucrose solution overnight, embedded in OCT compound (Tissue Tek; Qiagen, Valencia, CA, USA), and frozen in liquid nitrogen. With eyecups marked to maintain proper orientation, cross-sections 20 μm thick were obtained for each retina (CM1850 Cryostat; Leica, Wetzlar, Germany), collected on gelatine poly-L-lysine coated slides, and stored at −20 °C until processed. The analyses were performed on the sections crossing the optic nerve in the dorsal-ventral direction (~8–10 central sections per retina were collected on different slides) to minimize variations in the retinal length and position.

The morphological analysis was carried out by labeling the nuclei with the DNA-specific dye bisbenzimide and measuring the thickness of the outer nuclear layer (ONL) across the entire retinal extension from dorsal to ventral crossing the papilla, following a previously described procedure [40]. Shortly, histological reconstructions were obtained by joining consecutive acquired micrographs (each long ~380 μm, usually 10–12 per hemiretina). To facilitate comparison between retinas, each retina was divided into 10 dorsal and 10 ventral fields while taking the optic nerve as a reference. Analyses included: (1) the ratio of ONL to the total retinal thickness to measure ONL thickness (rather than the absolute thickness of the ONL (μm), to compensate for possible oblique sectioning); (2) the number of photoreceptor rows in the ONL; (3) damaged ONL length. ONL thickness and ONL rows were calculated as the averages of 4 equi-spaced measurements from each of the 20 fields (to account for slight differences within the same area). The averages of the ONL thickness and rows measured in the 10 dorsal or ventral fields were calculated for each section. We then evaluated the extent of damage in the photoreceptor layer by calculating the ratio between “damaged ONL length” (clearly detectable because of the presence of “rosette” structures [41]) and the total dorsal retinal length. Measurements were performed using ImageJ 2.0 software (Rasband, W.S., ImageJ, U. S. National Institutes of Health, Bethesda, MD, USA).

Together with nuclear labeling, the slides were immunolabeled with antibodies according to Table 2. Bovine serum albumin (BSA) (Sigma-Aldrich, St. Louis, MO, USA) or specific sera were used to block nonspecific binding sites, and Triton X−100 (Sigma-Aldrich) was used as a detergent, when appropriate. Primary antibodies were omitted to control for nonspecific binding of the secondary antibody. Only one of the two eyes was used for a single marker or histological analyses; therefore, the number of analyzed sections is equivalent to the number of animals (consequently, in figure legends, we reported N. as the number of analyzed retinal slices).

### 2.5. Microscopy and Image Analysis

Images of the retina were acquired with Leica TCS SP5 (Leica Microsystems, Wetzlar, Germany) or Nikon80i (Nikon, Tokyo), and acquisition parameters were kept constant throughout each imaging session. Measurements of fluorescence intensity and signal localization were made with ImageJ Fiji software (National Institutes of Health, Bethesda, MD, USA).

The L/M opsin signal was calculated following the method used by Rutar et al. [41]. Briefly, ten images (5 dorsal and 5 ventral) equally spaced were taken under 20× magnification from a total of 5 or 6 retinal sections from different rats for each experimental condition. We counted external outer segments (OSs) of cone photoreceptors. The number of positive cells is expressed as a mean of labeled cells per field or as their sum, respectively, for the dorsal and ventral retina.

Spatial distribution and the total number of Iba1^+^ microglial cells were analyzed in the ganglion cell layer and inner plexiform layer (GCL + IPL), in the inner nuclear layer, and outer plexiform layer (INL + OPL), and in the outer nuclear layer (ONL). Each retinal layer was determined according to the nuclear staining. The number of Iba1^+^ cells was manually counted throughout the entire section from dorsal to ventral (10 images per hemisphere acquired at 40× magnification, each micrograph covered ~380 μm of retinal length). Results are given as the total number of microglial cells (Iba1^+^) per layer and per section.

Densitometric analysis of GFAP and bFGF-2 fluorescent signals (*N* = 3–6 and *N* = 5–7 retinal sections, quantified in Figures 3 and 7, respectively) was performed using custom-written algorithms in ImageJ Fiji software (NIH, Maryland, USA). The percentage of areas positive for bFGF-2 or GFAP over the total area of the ONL or the entire retinal area, respectively, was determined by applying a threshold and calculated as described in Antognazza et al. [44].

Cholinergic neurons in the retina, known as starburst amacrine cells (SBAC), were labeled with anti-ChAT antibody. The integrity of their processes, located in the inner plexiform layer (IPL), was quantified in two areas of the retina (dorsal and ventral, respectively) relatively to the total length (*N* = 5 retinal sections from different rats for each experimental condition; HC and 24 h light-exposed rats). Cell bodies density was also quantified (data not shown because of lack of statistical significance).

Synaptophysin, a protein expressed in the presynaptic vesicles, was qualitatively assessed (acquired under 60x magnification) in 5 retinal sections for each experimental condition, and representative immunofluorescence images are shown in the figure.

### 2.6. Statistical Analysis

One or two-way ANOVA was used to evaluate the effects of different LD durations and recovery periods. When the significance level was 0.05 or less, post hoc pairwise comparisons were performed using Tukey’s test. Data are reported as mean ± standard error of the mean (SEM). Values of *p* < 0.05 were considered to be statistically significant. All statistical analyses were performed using Prism7 software (Graph Pad, San Diego, CA, USA). A detailed description of each test can be found in figure legends.

## 3. Results

### 3.1. Different Durations of Light Exposure: Discrepancies between Functional Output and the Extent of Structural Damage 

This section examines the effects of different light exposure durations (i.e., 12 h, 15 h, 18 h, 24 h at 1000 lux) on retina functional, morphological, and molecular states, assessed 7 days after the treatment. For the sake of simplicity, we refer to 12 h as short-term exposure (compared to 24 h).

#### 3.1.1. Exposure to Constant Light for 12 h Led to Functional but Not Severe Morphological Retinal Alterations

**Figure 1:**
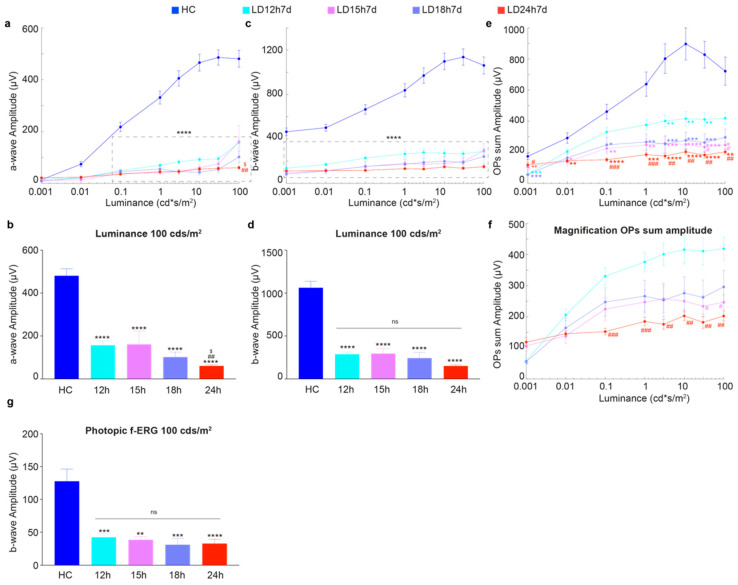
Retinal functional impairment following different durations of light exposure. (**a**,**c**) Amplitudes of the a-wave and b-wave in healthy control (HC) and different experimental groups (LD12h7d, LD15h7d, LD18h7d, and LD24h7d) are plotted as functions of stimulus intensity (0.001–100 cd*s/m^2^). A-wave amplitude in the HC was significantly higher than all groups exposed to light for all luminances brighter than 0.1 cd*s/m^2^. B-wave amplitude was significantly higher than all experimental groups at all luminances. (**b**,**d**) Panels b and d represent a-wave and b-wave amplitudes measured at 100 cd*s/m^2^. LD24h7d a-wave amplitude at 100 cd*s/m^2^ was significantly reduced compared to LD12h7d. ^##^ *p* = 0.0011, and LD15h7d, ^$^ *p* = 0.0177; two-way ANOVA with Tukey’s post hoc test. (**e**) Amplitudes of the OPs sum in HC and different experimental groups are plotted as functions of stimulus intensity (0.001–100 cd*s/m^2^). On average, OPs sum amplitudes were significantly affected by all durations of light exposure. (**f**) Panel f represents a large magnification of panel (**e**). The effect of light exposure was milder after 12 h; indeed, OP amplitude was significantly preserved in LD12h7d vs. LD24h7d at intensities brighter than 0.1 cd*s/m^2^. (**g**) The values represent the mean response amplitudes of photopic b-waves. Statistical significance is represented as follows: *, ^#^, ^$^ *p* ≤ 0.05, **, ^##^, ^++^ *p* ≤ 0.01, ***, ^###^ *p* ≤ 0.001, **** *p* ≤ 0.0001; *, ^#^, ^$^, ^+^ are in comparison to HC, LD12h7d, LD15h7d, and LD18h7d, respectively; two-way and one-way ANOVA with Tukey’s post hoc test were used to assess differences in the panels (**a**–**g**), respectively. Values represent means ± standard errors of the means (SEM). Between 6 and 12 retinas were recorded per group.

Figure 1 describes the effects of different exposure regimes on the retinal functional state, as assessed through in vivo flash electroretinography (fERG). Surprisingly, our analyses revealed no major functional differences between light damaged experimental groups, with a few exceptions. Indeed, even the short-term exposure induced a significant reduction of a-wave and b-wave amplitudes compared to the healthy control (Figure 1a,c). Interestingly, only the a-wave amplitude, obtained in response to the brightest stimulus (100 cd*s/m^2^), was better preserved in LD12h and LD15h compared to LD24h (Figure 1a,b for magnification; a-wave amplitude was ~2.5 times higher in LD12h and LD15h vs. LD24h, *p* = 0.0011 and *p* = 0.0177 respectively). Instead, our evaluation did not show any significant difference between retinas exposed to light with regard to the b-wave amplitude; as expected, they were all different compared to the HC (Figure 1c,d; *p* < 0.0001 for all stimulus intensities). In particular, fERG b-wave amplitude decreased by 30% or 15% in rats exposed to 12 or 24 h, respectively, compared to HC (LD12h7d = 287 ± 26 μV and LD24h7d = 150 ± 13 μV vs. HC = 1061 ± 76 μV at 100 cd*s/m^2^).

It is worth highlighting that by studying oscillatory potentials (OPs), an indicator of the inner retinal activity [45], some differences were unexpectedly found between the light-exposed experimental groups. As observed for the other two analyzed ERG parameters, the light damage paradigm caused a significant reduction of OP sum amplitude at all stimuli intensities (i.e., to ~35%, ~32%, and ~44% respectively in LD15 h, 18 h, and 24 h groups compared to HC at 10 cd*s/m^2^; LD15h7d = 250 ± 32 μV, LD18h7d = 276 ± 48 μV and LD24h7d = 203 ± 15 μV vs. HC = 896 ± 100 μV, in Figure 1e), but with a few exceptions. Indeed 12 h led to a significant reduction of the OP sum compared to HC only at stimuli intensities between 3 and 30 cd*s/m^2^ (reduction by ~22% at 10 cd*s/m^2^; LD12h7d = 415 ± 45 μV and HC = 896 ± 100 μV). What was more interesting was the difference between the two groups LD12h7d and LD24h7d (Figure 1f for magnification; at light intensities higher than 0.1 cd*s/m^2^), revealing evidence for a different time course and extent of light-induced lesions that interests the inner retina.

In contrast, no differences between groups (except for the reduction compared to HC) were observed when cone functionality was assessed by photopic-ERG. Indeed, 7 days after 12 h, 15 h, 18 h, and 24 h of light damage, the photopic b-wave amplitudes represented ~33%, ~30%, ~24%, and ~26% of the HC one, respectively (LD12h7d = 42 ± 5 μV, LD15h7d = 38 ± 5 μV, LD18h7d = 31 ± 10 μV, LD24h7d = 33 ± 7 μV and HC = 128 ± 18 μV; all significant vs. HC, Figure 1g).

#### 3.1.2. Outer Nuclear Layer (ONL) Disruption Does Not Correlate with Detectable Functional Alterations

**Figure 2:**
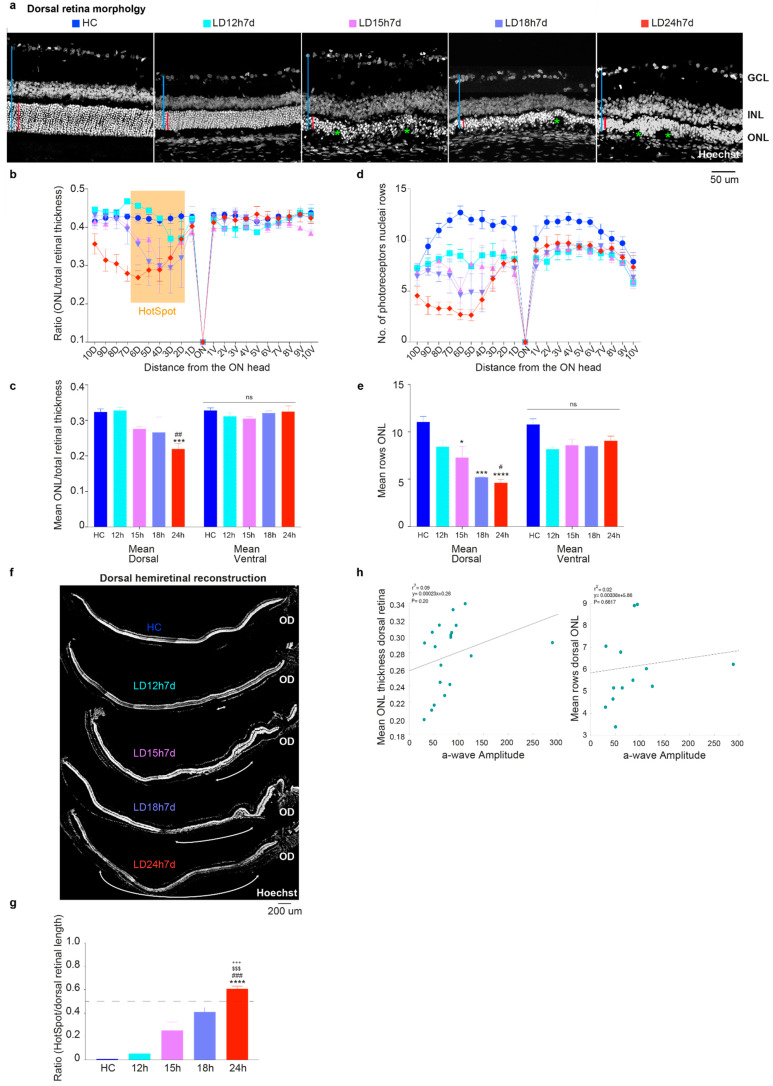
Morphometric evaluation of the light damage resulting from different exposure times. (**a**) Photomicrographs of the hot spot in the dorsal central retina of an undamaged control rat (HC) and rats exposed to 12 h, 15 h, 18 h, and 24 h of constant light; nuclei are stained with Hoechst. The red bars show the absolute thickness of the ONL, and the blue one the entire retinal thickness. Scale bar = 50 µm. Green asterisks signify disruptions in the ONL. Abbreviations: GCL: ganglion cell layer; INL: inner nuclear layer; ONL: outer nuclear layer. (**b**,**d**) ONL thickness and number of rows of photoreceptor nuclei (±SEM) quantified in 20 equidistant retinal locations (10 dorsal, 10 ventral). (**c**,**e**) Average across all dorsal or ventral retinal locations of the ONL thickness (ratio ONL/total retinal thickness) and the number of photoreceptor nuclei rows. (**f**) Representative dorsal hemiretina reconstruction after nuclei staining. The white arrows depict the damage extensions defined as “hot spots” on light damaged retinas. Scale bar = 200 µm. OD: optic disc. (**g**) Damaged ONL length/dorsal retina length for different light damage durations. (**h**) Relationship between a-wave amplitudes and ONL thickness/rows (Pearson correlation). Data in all graphs are shown as mean ± standard error of the mean (SEM). Statistically significant differences are represented as follows: *, ^#^ *p* ≤ 0.05, ^##^ *p* ≤ 0.01, ***, ^###^, ^$$$^, ^+++^ *p* ≤ 0.001, **** *p* ≤ 0.0001; *, ^#^, ^$^, ^+^ compared to HC, LD12h7d, LD15h7d, and LD18h7d, respectively; one-way ANOVA with Tukey’s post hoc test. *N* = 3/12 retinas from different rats for each experimental condition.

Figure 2a indicates that exposure to high intensity light for different durations of time (from at least 12 h to 24 h) caused a gradual alteration of the photoreceptor layer integrity in the dorsal retina, which has extensively been shown to be the more susceptible to induced neuronal death [41,46]. To analyze tissue structure, the cross-sectioned retinas, from the dorsal to ventral quadrant, were stained with Hoechst and the ONL thickness relative to the entire retina, and the number of nuclei rows in the ONL were analyzed. In the representative retinal sections in Figure 2a, we observed that the ONL underwent a reduction in thickness and structural changes correlated with the light exposure duration. Surprisingly, despite showing no apparent signs of retinal damage (Figure 2a, second micrograph) and a preserved ONL/retinal thickness ratio (Figure 2b,c; cyan, LD12h7d), the number of photoreceptor nuclei rows was slightly reduced (not significant) in the dorsal retina following 12 h of light exposure (Figure 2d). It is plausible to think that the loss of a limited number of photoreceptors does not change the ratio of ONL/total retinal thickness; indeed, the photoreceptor nuclei rows count may be a more convenient measure of mild damage, though does not account for possible oblique sectioning. As expected, the increase in light exposure duration produced a more significant thinning of the outer retinal layers, specifically in the hot spot area in the dorsal hemiretina (Figure 2c; LD24h7d significantly different compared to HC and LD12h7d, *p* = 0.004 and 0.0038, respectively).

In contrast, a significantly reduced number of nuclei rows compared to the HC was observed within the dorsal hemiretina also in the LD15h7d and LD18h7d groups (Figure 2e; LD15h7d, LD18h7d, and LD24h7d vs. HC, *p* = 0.0136, 0.0006 and <0.0001). This result is also significant when comparing LD24h7d to LD12h7d (respectively 4.6 vs. 8.4 rows, *p* = 0.0104). ONL disruption was then correlated with the a-wave amplitude (photoreceptors driven), and no correlation was found (Figure 2h, *p* = 0.20, and *p* = 0.6617 for the ONL thickness and N. rows vs. a-wave amplitude, respectively. Only 19 (LD12h7d = 5, LD15h7d = 4, LD18h7d = 4, and LD24h7d = 6) and 13 retinas (LD12h7d = 3, LD15h7d = 3, LD18h7d = 3, and LD24h7d = 4), for which both functional and morphological features were assessed, were included in this analysis. No HC was included to study the effect of light exposure). This result demonstrates that ERG prematurely diagnoses ONL matrix disorganization. Further analyses have been performed to measure the hot spot extension, evaluated as damaged ONL length/dorsal retinal length. Figure 2f presents a histological reconstruction of the dorsal hemiretina central section, with retinal damage extensions labeled with white arrows. We observed that the hot spot length increased with light exposure durations, covering up to 60% of the dorsal retina in the LD24h7d (Figure 2g).

#### 3.1.3. Upregulation of GFAP and bFGF-2 Was Triggered after Short Periods of Constant Light Exposure

**Figure 3:**
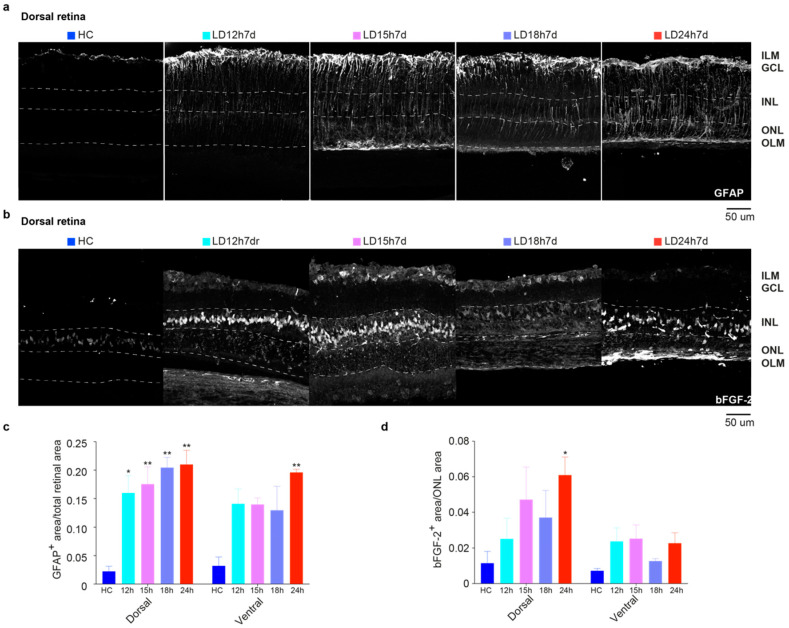
GFAP and bFGF-2 expression in retinas of rats exposed to light for different durations of time. (**a**) Representative photomicrographs illustrating GFAP^+^ astrocytes in dorsal retinal sections of HC and 7 days after 12 h, 15 h, 18 h, and 24 h of light damage (in the area around the degenerating hot spot, defined “penumbra” [41]), respectively. Light exposure induced the upregulation of GFAP in the radially oriented Müller cells; the protein was visible along the full length of the Müller cells, from the ILM to the OLM. (**b**) Representative photomicrographs illustrating bFGF-2^+^ signal in the dorsal retinal areas of control and light-exposed rats. Light exposure induced the upregulation of bFGF-2, released in the ONL by Müller cells; the protein is visible around photoreceptor bodies in the ONL and Müller cell bodies in the INL. Abbreviations: GCL: ganglion cell layer; INL: inner nuclear layer; ONL: outer nuclear layer; ILM: inner limiting membrane; OLM: outer limiting membrane. Scale bar = 50 μm. (**c**) Mean GFAP^+^ area averaged across dorsal and ventral retinal selected areas. (**d**) Mean bFGF^+^ area/ONL area averaged across the dorsal and ventral retina. Statistical analyses were performed with the analysis of variance (one-way ANOVA) followed by a Tukey’s post hoc test; one-way ANOVA with Trend was also used to analyze data in panel d. Statistically significant differences are represented as follows: * *p* ≤ 0.05, ** *p* ≤ 0.01, respectively, compared to HC. Data are shown as the means ± SEM of at least *N* = 3 retinas from different rats for each experimental condition.

Figure 3 describes a gradual increase in the expression of stress (glial fibrillary acidic protein, GFAP) and trophic markers (basic fibroblast growth factor-2, bFGF-2) in the retina, linked to light exposure. Under normal conditions, astrocytes and Müller glia contact retinal neurons, providing stability to the neural tissue. Stress of any origin induces an astrocyte response with increased expression of GFAP in the radially oriented Müller cells (MC). GFAP expression was confined to the GCL layer in the HC retinal section to form a homogeneous plexus (Figure 3a, first micrograph). Different light damage protocols induced upregulation of GFAP: the protein was visible along the entire length of MCs. To evaluate the effect of different light exposure durations, corresponding retina areas (one in the dorsal and one in the ventral) were selected to determine the relative GFAP-labelled regions [47]. For this purpose, the images were processed with ImageJ software’s threshold tool (see Materials and Methods). Areas of the retina marked with the threshold overlay (GFAP^+^ astrocytes plus GFAP^+^ end-foot of the MC; Figure 3a) were included in the measurement among study groups. Results obtained at 7d following 12 h, 15 h, 18 h, and 24 h light damage are shown quantitatively in Figure 3c. From the one-way ANOVA, performed separately for the dorsal and ventral retina, significant differences were found in the dorsal retina of all light-exposed groups compared to the HC (*p* = 0.0155, 0.0078, 0.0023, and 0.0018 for LD12,15,18, and 24 h vs. HC, respectively). Hence, even 12 h of light exposure was sufficient to produce retinal stress, in accordance with the ERG results. The upregulation of GFAP also extended in the ventral retina, which was significant only for the LD24h7d group compared to the HC (*p* = 0.0048). As expected, stressed retinas also increase the expression levels of trophic factors, such as bFGF-2. Notably, the release of bFGF-2 in the ONL negatively correlates with the b-wave amplitude [40]. In control retinas, bFGF-2 expression was confined to the Müller cell bodies in the INL (Figure 3b, first micrograph). Light damage induced its progressive release in the ONL (the protein was visible around photoreceptor cell bodies), and even though exclusively significant for the LD24h7d (*p* = 0.0288 vs. HC), it is noticeable that bFGF-2 levels increased in the dorsal retina after light exposure treatment. Indeed, a significant trend (*p* = 0.0168, one-way ANOVA with Trend) relates bFGF-2 release in the ONL to the duration of light exposure (no linear trend was revealed in the ventral side).

#### 3.1.4. Microglia Increased in the Dorsal Retina after Exposure to Constant Light

**Figure 4:**
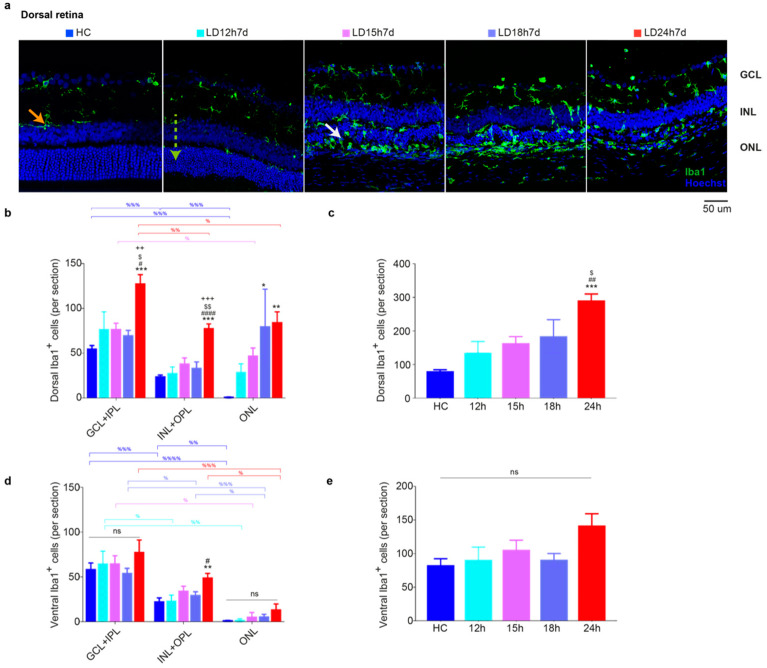
Microglia quantification in retinas of rats exposed to light for different lengths of time. (**a**) Representative photomicrographs illustrating Iba1^+^ cells in dorsal retinal sections (hot spot region) of HC and light-exposed retinas tested after 7 days. Light damage induced migration of resident Iba1^+^ cells to the ONL (significant after 18 or 24 h of light exposure). Orange arrows: ramified microglia; white arrows: ameboid shape; dashed green arrow: migration from the inner to the outer retina. Abbreviations: GCL: ganglion cell layer; INL: inner nuclear layer; ONL: outer nuclear layer. Scale bar = 50 µm. (**b**,**d**) Quantification of Iba1^+^ cells in dorsal and ventral retinal sections in each retinal layer. (**c**,**e**) Total numbers of microglia along the dorsal and the ventral retina (summarized from b, d data). Subretinal Iba1^+^ cells were not included in the count. Statistical analyses were performed with the analysis of variance (one-way ANOVA) followed by a Tukey’s post hoc test. Statistically significant differences are represented as follows: *, ^#^, ^$^ *p* ≤ 0.05, **, ^##^, ^$$^, ^++^ *p* ≤ 0.01, ***, ^+++^ *p* ≤ 0.001, ^####^ *p* ≤ 0.0001; *, ^#^, ^$^, ^+^ vs. HC, LD12h7d, LD15h7d, and LD18h7d, respectively; ^%^ was used to depict differences within layers. Data are shown as means ± SEM of at least *N* = 3 retinas from different rats for each experimental condition.

The immune response is triggered along with the degeneration of the outer retina and macrogliosis (assessed by anti-GFAP staining) [48]. Thus, we characterized the effect of light exposure on microglia distribution in the retina using Iba1 as a marker. Representative images of the dorsal retina from HC and the four experimental groups (different light exposure durations) are shown in Figure 4a (Iba1^+^ cells in green). As expected, [49], under physiological conditions, microglia that showed typical resting characteristics were located in the inner retina in HC (Figure 4b,d, GCL + IPL, and INL + OPL), and no cells were found in the ONL in the dorsal and ventral retina (GCL + IPL > ONL + OPL > ONL; *p* < 0.01). Exposure to light promoted their morphological change (from ramified to ameboid, depicted in Figure 4a with the orange and white arrows, respectively) and migration to the ONL (green dashed arrow in Figure 4a, LD12h7d) [50]. Detailed analyses of Iba1^+^ cells in the dorsal and ventral retina and across retinal layers are reported in Figure 4b–e. In the retinas of LD groups, we found that Iba1^+^ cells accumulate significantly in the ONL in the dorsal retina after 7 days from 18 h or 24 h of light exposure (LD18h7d vs. HC, *p* = 0.0013; LD24h7d vs. HC, *p* < 0.0001; one-way ANOVA). Overall, when the total number of microglia across all layers was assessed, a significant increase in Iba1^+^ cells was found only in the dorsal retina in LD24h7d compared to HC, LD12h7d, and LD15h7d (*p* < 0.0001, *p* = 0.0052, *p* = 0.0287, respectively). Only 24 h of light exposure induced a significant difference between the dorsal and ventral retina (*t*-test, LD24h7d dorsal vs. ventral, *p* = 0.04).

### 3.2. Long-Term Morpho-Functional Changes in the Neurodegenerative Progression

Given that 24 h of white light exposure resulted in well-established retinal structural and functional impairment after 7 days, we decided to investigate how the neurodegenerative process evolves using this experimental protocol. After inducing damage (24 h, 1000 lux), critical stages were determined within different retinal regions for up to 7 months.

#### 3.2.1. Timecourse Effects of 24 h of Bright Light Exposure Highlighted a Transient Functional Recovery

**Figure 5:**
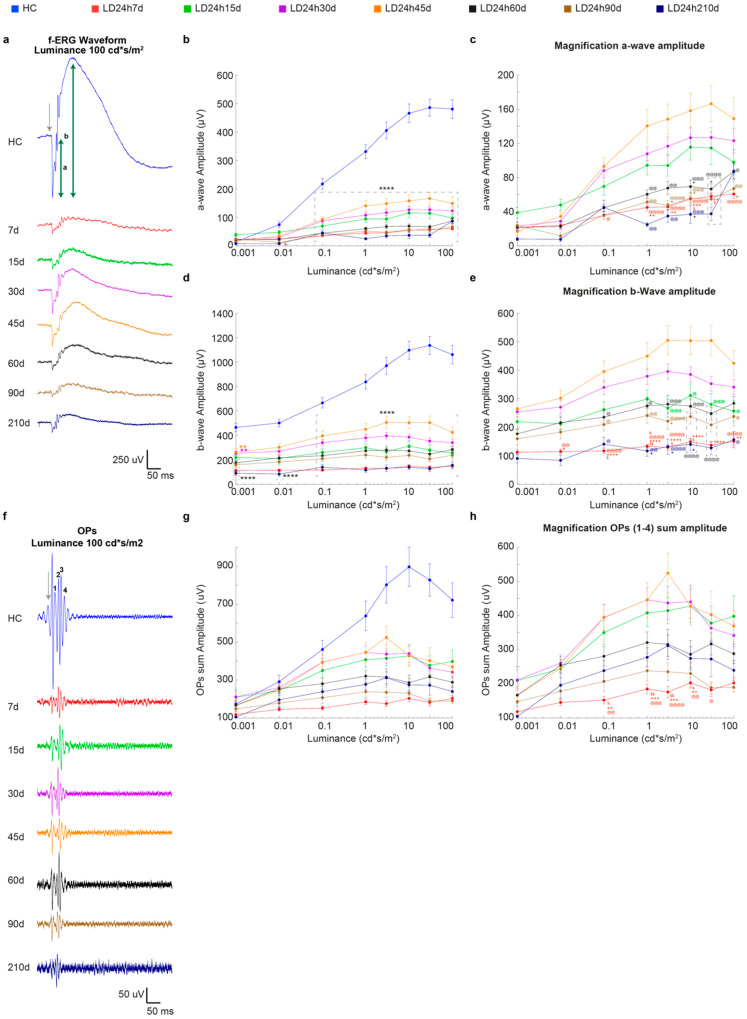
In vivo functional assessment at different recovery times following 24 h of light exposure. (**a**) Representative fERG waveforms in response to 100 cd*s/m^2^ flash intensity obtained from control and all experimental groups (7, 15, 30, 45, 60, 90, and 210 days following the end of light exposure). Calibration: horizontal 50 ms, vertical 250 μV. The grey arrow indicates when the flash stimulus was sent. (**b**,**d**) Amplitudes of the a-wave and b-wave in HC (blue line) and different experimental groups as a function of stimulus intensity (0.001–100 cd*s/m^2^). (**c**,**e**) represent magnifications of the graphs in (**b**,**d**). (**f**) Representative OP waveforms, isolated by bandpass filtering the dark-adapted ERGs to bright light (100 cd*s/m^2^), from HC and all experimental groups (7, 15, 30, 45, 60, 90, and 210 days following the end of light exposure). Calibration: horizontal 50 ms, vertical 50 μV. The grey arrow indicates when the flash stimulus was sent. 1–4 depict, respectively, OP1–4, whose amplitude was summed. (**g**) Amplitudes of the OP sum in HC (blue line) and different experimental groups as a function of stimulus intensity (0.001–100 cd*s/m^2^). (**h**) represents a magnification of the graph in panel (**g**). A single value represents the mean ± standard error of the mean (SEM). Statistical significance is represented as follows: *, ^$^, ^+^, ^@^, ^&^ *p* ≤ 0.05, **, ^$$^, ^++^, ^@@^ *p* ≤ 0.01, ^+++^, ^@@@^ *p* ≤ 0.001, ****, ^++++^, ^@@@@^ *p* ≤ 0.0001; *, ^$^, ^+^, ^@^, ^&^ represent comparisons with HC, LD24h15d, LD24h30d, LD24h45d, and LD24h60d, respectively; two-way ANOVA with Tukey’s post hoc test. The control group was significantly higher than all light damaged groups at all intensities higher apart 0.001 cd*s/m^2^ (only statistical results vs. HC are shown in (**b**,**d**,**g**); differences between light-exposed groups are shown in (**c**,**e**,**f**)). *N* = 15/30 recorded retinas per group.

**Figure 6:**
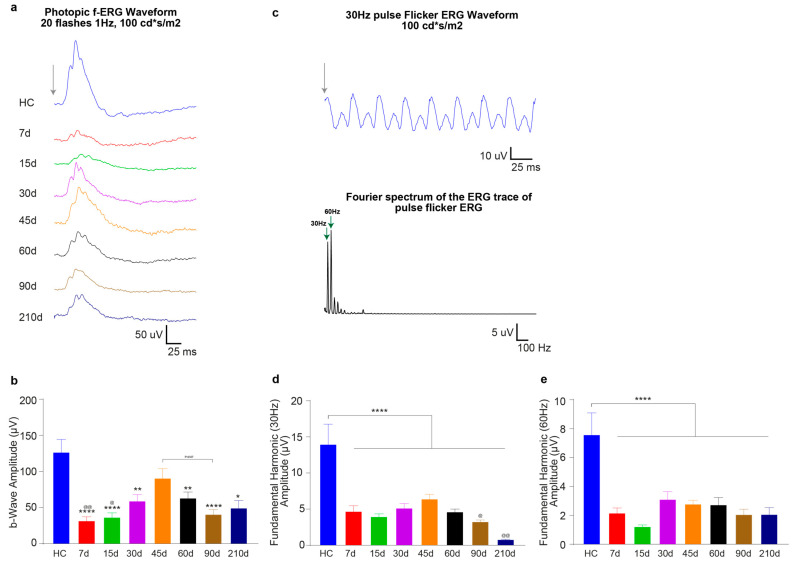
In vivo photopic functional assessment at different recovery times following 24 h of light exposure. (**a**) Representative averaged light-adapted ERGs in response to 20 bright flashes (100 cd*s/m^2^) of light at 1 Hz presented on a rod-saturating background. Calibration: horizontal 25 ms, vertical 50 μV. No stimulus baseline is included in the traces. (**b**) The values are the mean response amplitudes of photopic b-waves recorded from control animals before light exposure (HC) and 7, 15, 30, 45, 60, 90, or 210 days after 24 h light exposure. The photopic response is affected by light exposure but recovers almost completely by 45 days. Values represent mean ± standard error of the mean (SEM). (**c**) Representative photopic flicker response in control rats elicited at 30 Hz. Each waveform was recorded for 200 ms, and responses are the averages of 100 tests. (**d**,**e**) Amplitudes of the photopic flicker responses in LD rats are lower than those of the HC. Values represent mean ± standard error of the mean (SEM). Statistical significance is represented as follows: *, ^@^ *p* ≤ 0.05, **, ^@@^ *p* ≤ 0.01, **** *p* ≤ 0.0001; * and ^@^ are in comparison to HC and LD24h45d, respectively; one-way ANOVA with Tukey’s post hoc test. *N* = 15/30 recorded retinas per group.

We analyzed the time course of ERG response variations following 24 h of light-induced damage. Long-term monitoring of neurodegenerative processes underlined a transient increase in retinal function, followed by a further decline. Figure 5a depicts the response series of dark-adapted flash ERG recordings (at luminance 100 cd*s/m^2^, representative waveforms) before light damage (HC, age 60 days), and 7, 15, 30, 45, 60, 90, and 210 days after the light damage (24 h, 1000 lux). Quantitative analysis indicated a significant decrease of ERG components (a-wave and b-wave amplitudes) recorded at 7 days after light exposure and described a partial recovery, maximum at 45 days, followed by an irreversible drop. In fact, at 7d after LD, the dark-adapted a-wave maximum amplitude (at 10 cd*s/m^2^) decreased significantly compared to HC (LD24h7d = 55 ± 4 μV vs. HC = 466 ± 33 μV, *p* < 0.001) to ~12% of the pre-LD level (HC), and the b-wave maximum amplitude decreased to ~14% (HC = 1098 ± 74 μV and LD24h7d = 149 ± 10 μV, *p* < 0.001). Figure 5b–e indicates that by 45d, the dark-adapted a and b-waves maximum amplitudes (at 10 cd*s/m^2^) recovered ~34% and ~45% of their initial values (LD24h45d a-wave = 159 ± 21 μV, and b-wave = 503 ± 49 μV vs. HC = 466 ± 33 μV and HC = 1098 ± 74 μV). This amelioration was found to be transient: significant decrements of a-wave and b- wave amplitudes were evident at longer recovery periods (LD24h45d vs. LD24h60d, *p* = 0.001 and vs. 90d, and 210d, *p* < 0.001 with respect to the b-wave amplitude; and LD24h45d vs. LD24h60d, *p* = 0.001, vs. 90d, *p* < 0.001, and 210d, *p* = 0.0008). Figure 5f illustrates the filtered oscillatory potentials (OPs) from control and light-exposed retinas at different recovery times following the same light exposure protocol. The four analyzed OPs are enumerated on the HC trace (OP 1–4). Fifteen days were enough to detect significant partial recovery (LD24h7d vs. LD24h15d, *p* ≤ 0.05 at 0.1–10 cd*s/m^2^). By 45d the OP sum amplitude recovered to over 50% of the initial HC value and was significantly higher than LD24h7d (HC = 897 ± 100 μV; LD24h7d = 203 ± 15 μV vs. LD24h45d = 427 ± 53 μV at 10 cd*s/m^2^, *p* = 0.006). After 45d, the significant difference from LD24h7d was lost, meaning that OPs amplitudes diminished and deteriorated until 210d (Figure 5h). Light adapted responses (cone-driven activity) followed a similar pattern: at 7d after the light-damage, the LD24h7d average light-adapted b-wave amplitude reached only 26% of its baseline (HC = 128 ± 19 μV and LD24h7d = 33 ± 7 μV, *p* < 0.001), but recovered almost completely by 45 days (indeed LD24h45d = 92 ± 14 μV non-significant vs. HC, but significant vs. LD24h90d = 41 ± 7 μV, *p* = 0.07) (Figure 6b). Moreover, responses to brief flashes at 30 Hz were recorded (with flash intensities of 100 cd*s/m^2^). We analyzed the flicker ERG responses in the frequency domain (Figure 6c), and the averaged results are illustrated in Figure 6d,e. The two graphs illustrate the mean amplitudes of the fundamental and the second harmonics of the flicker responses at 100 cd*s/m^2^. Light exposure flattened the amplitude of the fundamental and the second harmonic in all experimental conditions. In addition, we observed a significant worsening of function at later stages of the neurodegeneration (indeed, the first fundamental harmonic amplitudes at LD24h90d and 210d were significantly reduced compared to LD24h45d; Figure 6d).

#### 3.2.2. The Ventral Retinal Structural Layer Organization Was Better Preserved Compared to the Dorsal One

**Figure 7:**
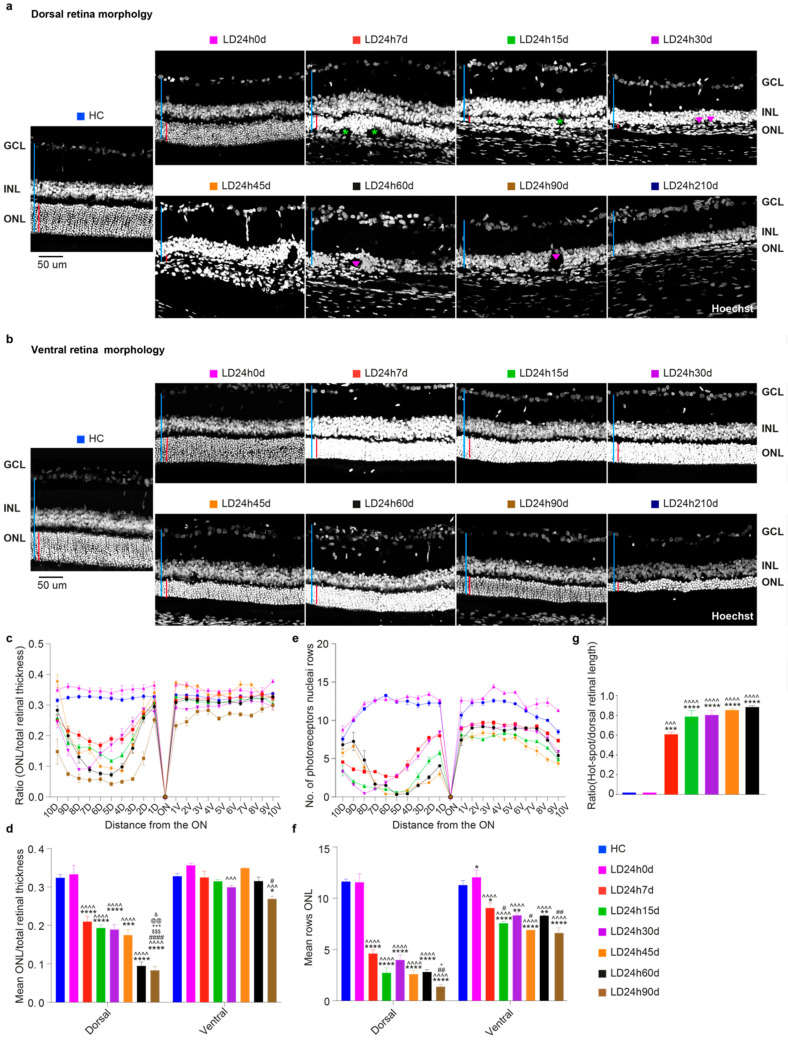
Morphological alterations were assessed at different recovery times following 24 h of light damage. (**a**,**b**) Representative Hoechst-stained retinal cross-sections from the dorsal and ventral central retina. Light damage was more severe in the dorsal retina, considering the matrix disorganization. Abbreviations: GCL: ganglion cell layer; INL: inner nuclear layer; ONL: outer nuclear layer. The red and blue vertical bars show the absolute thickness of the ONL and total retinal thickness, respectively. Green asterisks mark the rosette structures in the ONL, and pink triangles the holes in the INL. Scale bar = 50 μm. (**c**,**e**) Graphs showing the ONL thickness and number of photoreceptor nuclei rows (±SEM) in the ONL in 20 equidistant retinal locations (10 dorsal, 10 ventral). (**d**,**f**) The average and standard error of ONL thickness (ratio ONL/total retinal length) and numbers of photoreceptor rows. Photoreceptor loss (cell rows) in the retina was significant after 7 days of recovery and progressed for up to 7 months (210 days are not shown on the graph because it was hard to quantify these two parameters because of the tissue’s damage). (**g**) Damaged ONL length analyses for different recovery times. The extension of the damaged area significantly increased over time, never reaching the retina’s ventral side. Histograms show means ± SEM. Statistical significance is represented as follows: *, ^#^, ^&^, ^+^ *p* ≤ 0.05, **, ^##^, ^@@^ *p* ≤ 0.01, ***, ^^^^^, ^$$$^, ^+++^ *p* ≤ 0.001, ****, ^^^^^^, ^####^ *p* ≤ 0.0001; *, ^^^, ^#^, ^$^, ^+^, ^@^, ^&^ refer to HC, LD24h0d, LD24h7d, LD24h15d, LD24h30d, LD24h45d, and LD24h60d, respectively; one-way ANOVA with Tukey’s post hoc test. Values represent the means ± standard error of the mean (SEM) for at least *N* = 5 retinas from different rats for each experimental condition.

The loss of photoreceptors induced by 24 h light exposure accompanied a progressive decrease in the ONL thickness over time. Figure 7 displays the histological appearance of the dorsal (a) and ventral (b) retina along the vertical meridian from control rats and those exposed to 24 h of high-intensity light at different time-points following the light exposure. The dorsal retina was more severely affected than the ventral one, whose retinal layered structure appeared better preserved (no rosette structures or matrix disorganization). Photoreceptor nuclei were few and dispersed in the dorsal side. The INL directly neighboring the RPE, and several holes were present in the retina 30 days after light exposure (see also Figure 9a). The average ratios and the mean number of photoreceptor rows for all twenty quadrants (Figure 7d,f) also decreased in the ventral side in LD animals.

Furthermore, despite photoreceptor rows’ progressive reduction (Figure 7f; all LD groups are significantly different vs. HC), relative ratios in the ventral retina were conserved, underling a homogeneous thinning among retinal layers up to 60 days of recovery (mean ONL/total retinal thickness overall ns vs. HC till later stages of neurodegeneration). A further analysis was done to measure the extent of damage, evaluated as damaged ONL length/dorsal retinal length. In the central section, crossing the optic nerve, the amount of dorsal ONL damage increased over time, reaching 80% of the dorsal retina after 60 days (Figure 7g).

#### 3.2.3. Light-Induced Cone-Photoreceptor Damage

**Figure 8:**
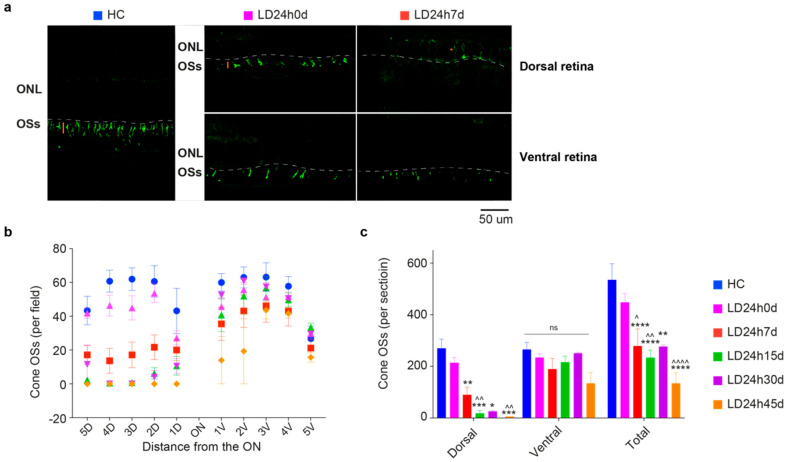
Light-induced damage to the L/M cone-photoreceptors population. (**a**) Retinal cross-sections from a representative HC, and 0 and 7 days after light-induced retinal damage. Sections are labeled with an antibody against L/M opsin. Outer segments (OSs) appear normally distributed throughout the retina in the control condition. Light-exposed retinas immediately showed a partial reduction of cone density (0 d) depicted by the length of the orange bar and redistribution of the signal into the soma (*, orange) in the dorsal retina, shortening the remaining OSs in the ventral at 7d. Abbreviations: ONL: outer nuclear layer; OSs: outer segments. Scale bar = 50 µm. (**b**,**c**) Numbers of cones distributed along the retinal sections passing through the optic nerve (ON), the totals on the dorsal and ventral sides, and their sum for HC, LD24h0d, LD24h7d, LD24h15d, LD24h30d, and LD24h45d. Statistical significance is represented as follows: *,^^^ *p* ≤ 0.05, **,^^^^ *p* ≤ 0.01, *** *p* ≤ 0.001, ****,^^^^^^ *p* ≤ 0.0001; * and ^^^ refer to HC and LD24h0d, respectively; two-way ANOVA with Tukey’s post hoc test. Data represent the means ± SEM of at least *N* = 5 retinas from different rats, for each experimental condition.

Following light exposure, the first sign of damage is observed at the level of photoreceptor OSs. Retinal sections were stained with an antibody for L/M opsin, allowing differentiation of individual cones for measuring cone density. L/M cones (in general also referred to as L-cones, because of the opsin they express) are mainly localized in the medial and central retina, where light exposure effects are more pronounced, and they are more abundant than S cones (densest in the retinal rims and periphery [51]). Figure 8a shows a composite of photographs taken from the control with uniform cone photoreceptor staining and light-exposed retinas characterized by a diminished density of cones and almost an absence of their corresponding external segments (cone OSs) on the dorsal side. As previously shown [41], the L/M opsin immunostaining revealed a sparse and disorganized population of surviving cones in the hot spot. After 7days, the dorsal retina’s remaining cones lost their normal elongated morphology and were immunoreactive to L/M opsin throughout the soma and axon terminals [52].

The remaining L and M-cones in retinal sections are plotted in Figure 8b,c. Twenty-four hours of light damage resulted in the loss of approximately 75% of the L/M-cone population by 45 days: detected cones were 134.3 ± 41.81 (*N* = 3) at 45 days compared to 535.40 ± 62.55 (*N* = 5) in the control (*p* = 0.0014) (Figure 8c). Sometimes, even though it was difficult to identify associated Hoechst-stained cell nuclei within the ONL, some residual L/M opsin immunoreactivity was present. Overall, in the ventral retina, no significant differences were found between HC and experimental groups up to the time point evaluated; a decrease was detectable only in the first two acquired fields (1- and 2-V) of ventral hemiretina at LD24h45d, in proximity to the damaged area (Figure 8b,c). Animals sacrificed after 45d were not included in cone density analysis. The number of lost cones progressed further from 7 to 45 days after light damage, without any significant rescue.

#### 3.2.4. Light Exposure Induced Modifications in the Inner Nuclear Layer (INL), Detectable at Least after 15 Days Following Light Exposure

**Figure 9:**
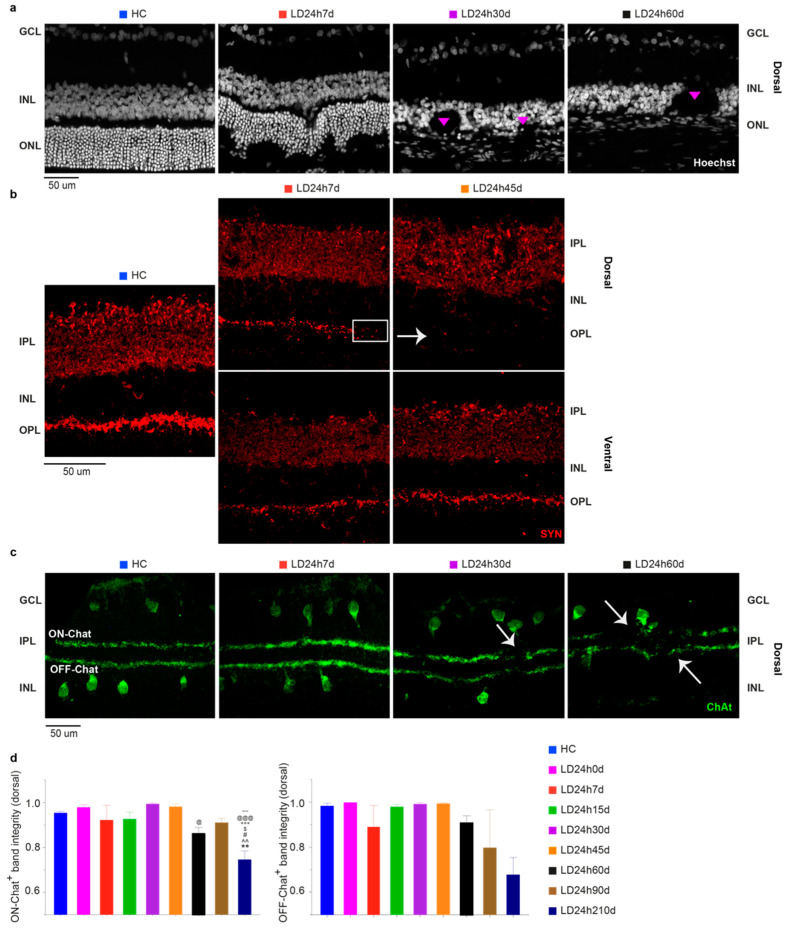
Inner retinal changes at different recovery times following 24 h of light exposure. (**a**) Hoechst-stained retinal cross-sections from the dorsal (hot spot) retina. A considerable disruption of the integrity of the INL is depicted. Structures like rosettes were formed (pink triangles) (see Figure 7b; INL holes were not present in the ventral retina). (**b**) Changes in synaptophysin (SYN) expression after 24 h light exposure were assessed on retinal cross-sections stained with an anti-SYN antibody. Compared to HC, there was a fainter expression in the dorsal OPL at 7 d (white rectangle) and almost no expression at 45 d (white arrowhead). (**c**) Retinal sections immunolabeled with anti-ChAT antibody revealed positive cell bodies in the IPL and GCL (OFF and ON Chat, respectively) and two narrowly stratified immunoreactive bands appearing in the IPL that presented interruptions from 30 days on after the light exposure (white arrowheads). (**d**) ON and OFF Chat bands were “integrity” normalized to the analyzed section length. Abbreviations: GCL: ganglion cell layer; IPL: inner plexiform layer; INL: inner nuclear layer; OPL: outer plexiform layer; IPL: inner plexiform layer; ONL: outer nuclear layer. Scale bar = 50 µm. Statistical significance is represented as follows: ^#^, ^$^, ^@^ *p* ≤ 0.05, **, ^^^^, ^~~^ *p* ≤ 0.01, ^+++^, ^@@@^ *p* ≤ 0.001; *, ^^^, ^#^, ^$^, ^+^, ^@^, ^~^ refer to HC, LD24h0d, LD24h7d, LD24h15d, LD24h30d, LD24h45d, and LD24h90d, respectively; one-way ANOVA with Tukey’s post hoc test. Data are shown as means ± SEM of *N* = 5 retinas from different rats for each experimental condition in panel d.

Morphological analyses mostly underlined a longitudinal extension of the hot spot area. However, as previously described from the ratio analysis (ONL thickness/total retinal thickness) and in other studies [28,29,34,53], the inner retina is also severely implicated. Figure 9a illustrates representative nuclei-stained retinal cross-sections after 7, 30, and 60 days post light exposure treatment. Gradually, with increasing time (after 7 days), the INL structure appeared highly disorganized, considerably thinned, and characterized by the presence of hole-like areas (Figure 9a; see also Figure 8a,b for a comparison between dorsal and ventral). Furthermore, in light of our functional data indicating a partial recovery of the a-wave and the b-wave, peaking at 45 days, we speculated that there could be compensatory retinal changes to account for the functional recovery. To assess this possibility, we studied the expression pattern of synaptophysin (SYN), an integral membrane protein expressed in presynaptic vesicles. In control retinas, SYN was expressed in the IPL and OPL, whereas after light exposure, the OPL signal was fainter compared to control (Figure 9b). Accordingly, we looked for other biochemical and immunohistochemical changes in the inner retina. Therefore, we aimed to establish whether neuronal modifications in the inner retina occurred in the light damaged rat model using an amacrine-specific marker. We stained retinal sections with an antibody against choline-acetyltransferase (ChAT), revealing the so-called ON and OFF starburst amacrine cells. These are critical elements in the encoding of motion, heavily contributing to direction-selective ganglion cells properties. We observed that although there was no reduction in the number of marked cell bodies either in the INL or GCL (data not shown), their dendritic arborization was disrupted 30 days after light damage (Figure 9c,d; significant at 210d vs. all experimental groups assessed at an earlier time point). As with other markers, ChAT expression in the ventral retina was mainly unaffected by neurodegenerative progression (data not shown).

#### 3.2.5. Spatial Differences in Glial Cells’ Reaction, bFGF-2, and Microglia Modulation: Non-Neuronal Cells’ Involvement in Retinal Degeneration

**Figure 10:**
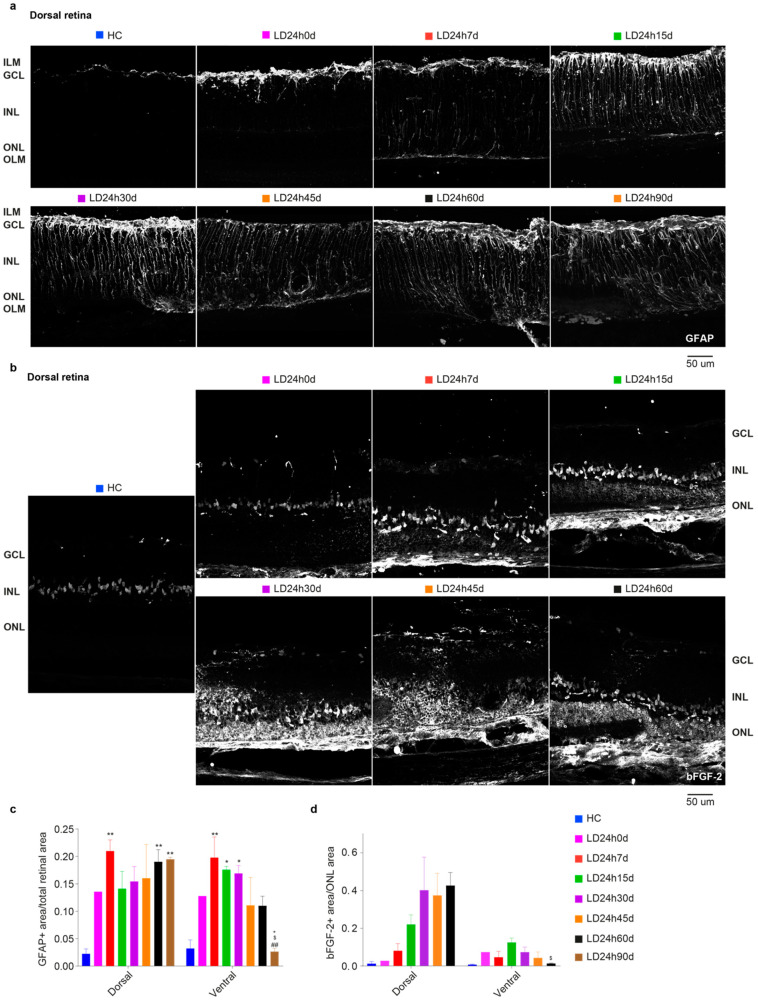
Distribution and density of GFAP and bFGF-2 in light-exposed retinas at different recovery times following 24 h of light damage. (**a**) Representative photomicrographs illustrating GFAP^+^ astrocytes in dorsal retinal sections (penumbra, around the degenerating hot spot) of control retinas and retinas monitored several days after light exposure. Light exposure triggered an increased expression of GFAP in the radially oriented Müller cells; the protein was visible along the full length of the Müller cells, from the ILM to the OLM. (**b**) Representative photomicrographs illustrating bFGF-2^+^ cells in dorsal retinal sections (penumbra) of control and light-exposed retinas were assessed several days after. Light exposure increased the expression of bFGF-2 released in the ONL by Müller cells; the protein was visible around photoreceptor bodies in the ONL and Müller cell bodies in the INL. (**c**) Mean GFAP^+^ area (retinal area occupied by GFAP^+^ signal) averaged across the dorsal and ventral retina. (**d**) Mean bFGF^+^ area (ONL area occupied by bFGF^+^ signal) averaged across the dorsal and ventral retina. Abbreviations: GCL: ganglion cell layer; INL: inner nuclear layer; ONL: outer nuclear layer; ILM: inner limiting membrane; OLM: outer limiting membrane. Scale bar = 50 µm. Statistical significance is represented as follows: *, ^$^, ^+^ *p* ≤ 0.05, **, ^##^ *p* ≤ 0.01; *, ^#^, ^$^, ^+^ refer to HC, LD24h7d, LD24h15d, LD24h30d, respectively; one-way ANOVA with Tukey’s post hoc test. One-way ANOVA with Trend was also used to analyze data in panel (**d**). Data are shown as means ± SEM of at least *N* = 5 retinas from different rats for each experimental condition in panel (**d**).

Light-induced gliosis was assessed by studying the evolution of astrocyte alterations during retinal degeneration through immunocytochemical localization of GFAP. Figure 10a illustrates the representative distribution of the GFAP signal in the marginal hot spot area (penumbra [41]). Due to the highly disorganized structure typical of the chronic phase, GFAP expression measurements were performed in the penumbra area. Quantification of the signal across all retinal layers (Figure 10c) discloses a significant increase in GFAP immunoreactivity after 7 days both on the dorsal and ventral sides compared to the control. Anyway, in the ventral retina, whose structure was better preserved, the GFAP^+^ area peaked at 30 days. It slightly decreased by 45 days, with significant differences between LD24h7d, 15d, and 30d vs. LD24h90d (one-way ANOVA, Tukey’s test, *p* = 0.0067, 0.0192 0.0265, respectively). Notice that at 90d GFAP^+^ signal in the dorsal retina was significantly higher compared to the ventral retina (*p* = 0.0032).

**Figure 11:**
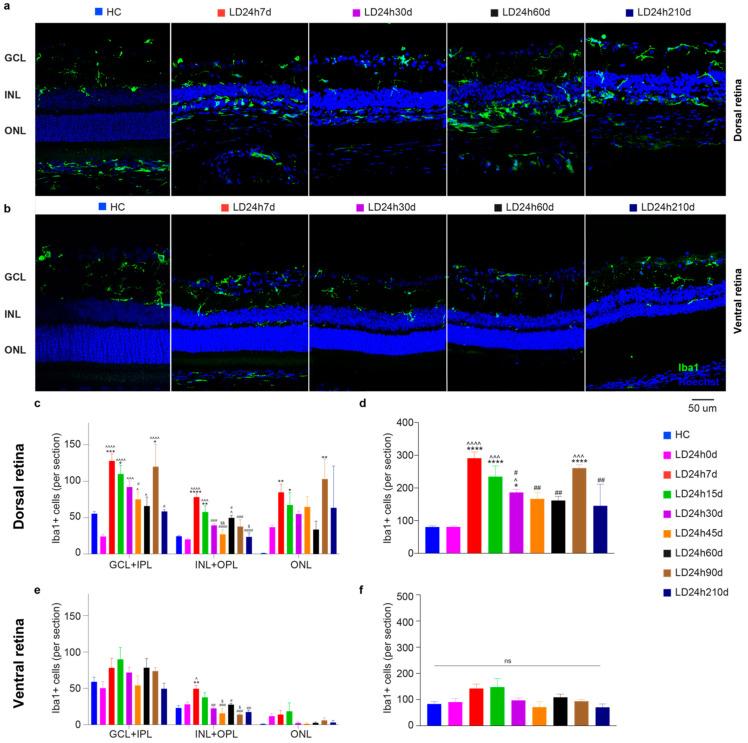
Quantification of microglia cells at different recovery times after 24 h of light exposure. (**a**,**b**) Representative photomicrographs illustrating Iba1^+^ cells in dorsal retinal sections (hot spot) of control and light-damaged retinas after light exposure. Light damage increased numbers of Iba1^+^ cells in the ONL by 7 d in the dorsal retina. Abbreviations: GCL: ganglion cell layer; INL: inner nuclear layer; ONL: outer nuclear layer. Scale bar = 50 µm. (**c**–**f**) Total numbers of microglia in the dorsal and ventral retina in each retinal layer (**c**,**e**) and summed across the entire retinal thickness (**d**,**f**). Statistical significance is represented as follows: *, ^^^, ^#^, ^$^ *p* ≤ 0.05, **, ^##^, ^$$^ *p* ≤ 0.01, ***, ^^^^^, ^###^ *p* ≤ 0.001, ****, ^^^^^^, ^####^ *p* ≤ 0.0001; *, ^^^, ^#^, ^$^ refer to HC, LD24h0d, LD24h7d, LD24h15d, respectively; one-way ANOVA with Tukey’s post hoc test. Data are shown as mean ± SEM of at least *N* = 5 retinas from different rats for each experimental condition.

On the contrary, in the penumbra (dorsal retina), glial cell reaction was less exacerbated at 15 d, 30 d, and 45 d than at 60 d and 90 d. Müller cells’ support to photoreceptors was monitored by analyzing the distribution and the protein level of bFGF-2 in the ONL using immunohistochemistry, at successive survival times after 24 h LD (Figure 10b). No significant differences among groups were detected, although bFGF-2 expression was linearly augmenting in the dorsal retina up to 60d (Figure 10d; one-way ANOVA with Trend, *p* = 0.0099. Non-significant trend in the ventral side was found, *p* = 0.2592). On the ventral side, bFGF-2 expression increased immediately after LD, became prominent at about 15 d, and then decreased to control levels by 60d (indeed LD24h60d dorsal is significantly different compared to LD2460d ventral). The immune response also accompanies neurodegeneration for a considerable time. The onset of photoreceptor degeneration activated the recruitment of retinal microglia from the plexiform layer to the injured site (see Figure 3). The analysis of Iba1^+^ cells distribution among layers described a consistent migration of resident microglia from the inner retina (GCL-IPL and INL) to the ONL, which required more than 24 h to occur (indeed LD24h0d not significant vs. HC). Iba1^+^ cells (both microglia and macrophages) decreased in the inner retina during more extended recovery periods from the onset of neurodegeneration and increased in the ONL. Following the initial typical acute neuroinflammatory response, from 30 to 60 days, we noticed a significant decrease of microglial cells in the dorsal retina (Figure 11d). No differences were detected in the ventral retina when microglia cells were summed across layers (Figure 11f).

## 4. Discussion and Conclusions

Most neurodegenerative retinal diseases are due to progressive degeneration of rods or cones. Current treatment strategies and paradigms manage to slow down photoreceptor loss or replace them using prosthetic devices or stem cells [6,7,8,9,11,12]. The therapeutic efficacy of these approaches depends on the diagnosis of the disease, the timing of which is usually related to the symptom’s occurrence. At later neurodegenerative stages, however, therapeutical approaches are less effective. Moreover, early dysfunctions of retinal diseases might be overcome by compensatory mechanisms of the retina, which could silence pathological signs [54,55]. Therefore, it is paramount to achieve rapid diagnosis and effective, targeted treatment at the earliest possible stage.

Aiming to understand better the critical steps involved, we exposed adult albino rats to bright light to induce retinal neurodegeneration and followed the disease’s morphological and functional progression. It was previously shown that this model mimics some aspects of human retinal degenerative disorders [56]. Our experiments first goal was to determine the minimal duration of bright light exposure necessary to induce retinal damage. Likewise, the second goal was to study the pathophysiological changes of the diseased retina over time, extending the focus to the inner retina.

### 4.1. Different Durations of Bright Light Exposure Provided Insights into Early Events in the Pathology of Light-Induced Retinal Damage

The first aim of our experiments was to characterize retinal pathophysiological processes following different durations of light exposure. We revealed that 12 h of homogeneous light exposure is sufficient to severely reduce the retinal response to light (assessed by in vivo analyses). However, this was not associated with critical morphological changes. Indeed, functional changes precede anatomical ones.

Specifically, the dark-adapted response is more severely impaired than the light-adapted response, in agreement with the higher survival rate of cones compared to rods, confirming previous studies about the etiology (rhodopsin-mediated [57,58]) of this light-induced retinopathy [59]. Interestingly, OP sum amplitude analysis revealed that the deterioration of OPs correlated better with the duration of light exposure than did the amplitudes of the a-wave and b-wave. The OPs are described as low-voltage, high-frequency components of the ERG [45] and are believed to reflect the synaptic activity of inhibitory feedback processes generated mainly by the amacrine cells. However, contributions of other inner retinal cells cannot be excluded [45]. Our findings indicated that 12 h of bright light exposure did not strongly affect the inner retinal circuitry, as suggested by the more preserved OP amplitude at LD12h7d (at least at specific stimuli intensities) despite continuing a-wave and b-wave amplitudes reduction.

Remarkably, by analyzing morphological features, we demonstrated that a minimum of 15 h is required to trigger the development of a hot spot (matrix disorganization and rosette structures formation) in the dorsal retina at 7 days after damage [41], which characterizes the model of light damage. Conversely, retinal morphology seemed better preserved after 12 h of light exposure, given the more subtle changes in photoreceptor rows and microglia numbers. Aside from the absence of a prominent hot spot in the LD12h7d group, the functional response (a-wave and b-wave amplitude) was similar to those of all other groups. Therefore, histological results at the ONL level did not correlate with those achieved at the functional level. Given this, we examined how different mechanisms might occur even before ONL disorganization and could influence the functional in vivo test. One such factor, the cytoskeletal type III intermediate filament protein GFAP of astrocytes, is considered an early marker for retinal injury and is commonly used as an index of gliosis-hypertrophy [60,61]. Twelve hours of exposure to light was enough to activate Müller cells, which are well-known to control several retinal trophic factors [62]. Simultaneously, activation of self-protective mechanisms might impinge on the retinal response to light, as supported by studies showing that an increase in bFGF-2 causes a reduction of the b-wave [40,63]. Moreover, it was interesting to notice that while the architecture of the ventral retina is affected more moderately than the dorsal retina (the layered structure of the retina was maintained), the inflammatory markers are upregulated compared to their baseline values. The upregulation of a range of protective factors might contribute to photoreceptor stability and protection; however, this does not extend to retinal performance, as ERG reduction shows.

This study disclosed that 12 h of light damage was sufficient to elicit clear signs of retinal dysfunction with minor morphological signs of damage in the dorsal hot spot area. For some parameters, such as photopic ERG, the functional impairment was irrespective of the exposure duration. Overall, this indicates that functional alterations precede morphological ones. These findings could be used to develop a diagnostic test that highlights an inflammatory condition in the retina that impinges on retinal functional output, as demonstrated. Indeed, ERG responses, recorded with an active extracellular electrode, according to Ohm’s law, might be affected by a change in the resistance. We are currently investigating whether neuroinflammation can bias the ERG output by changing the resistance of the eye. Consequently, the substantial initial reduction in retinal function is likely due to both electro-mechanical problems and the neuroinflammatory state that causes a change in tissue resistances.

### 4.2. Analyses Performed after Different Recovery Periods Following 24 h of Bright Light Exposure Showed Discrepancies between the Morphological and Functional Timecourses of the Disease

To achieve our second goal, retinas exposed for 24 h to light at 1000 lux were analyzed at different time points after the cessation of light exposure (acute phase)—up to 7 months (chronic phase). This study allowed us to categorize the pathophysiological process at different stages. The immediate consequence of light exposure was characterized by a progressive and irreversible loss of photoreceptors and a significant attenuation of retinal function in response to light. The chronic phase was characterized by a significant partial recovery, maximal at 45 days, despite the hot spot region gradually enlarging. Our findings suggest that during this stage, the retina attempted to recover from the light insult. Functional rescue following a phototoxic insult is a well-known phenomenon in light-damaged rats [36,64]. It was shown by Rutar et al. [41], as well as others, that once the animal is returned to standard lighting conditions, regrowth of the outer segment occurs, although we found a reduction in the total number of cones expressing L/M-opsin up to 45 days after light exposure. Schremser and Williams [65] showed that growing outer segments to their maximal length could take 20 days, but our study observed the maximal ERG amplitude value at 45 days. While the a-wave’s (linked to photoreceptor function) maximum amplitude recovered to ~34% (LD24h45d a-wave = 159 ± 21 μV and HC = 466 ± 33 μV, at 10 cd*s/m^2^) [66], the b-wave’s (second-order neurons and Müller cells) amplitude recovered to ~45% of its pre-LD value (LD24h45d b-wave = 503 ± 49 μV and HC = 1098 ± 74 μV, at 10 cd*s/m^2^). Conversely, OP amplitudes recovered significantly (~50% at 10 cd*s/m^2^), indicating a more pronounced rescue of the inner retina at 45 days.

We recorded ERG responses for up to 7 months and observed that retinal function deteriorates again after 45 days. As already described, astrocyte retinal response is triggered after bright light exposure and is followed by an upregulation of neurotrophic factors [67]. We demonstrated that after 24 h of light damage, the retinas of adult rats enhanced the expression of GFAP. This increase was maintained for up to 7 days in the dorsal side and up to 30 days in the ventral, and there was a subsequent ventral reduction at 45 days (ONL structure was more preserved) that became significant at 90 days, suggesting that GFAP may contribute, along with bFGF-2, to improving retinal function. bFGF-2 expression increased over time (significant trend) in the dorsal ONL for up to 60 days, but in the ventral side, its increase started immediately after LD and became prominent at approximately 15 days. Subsequently, it declined to control levels by 60 days (indeed LD24h60d significantly different only compared to LD24h15d). This slower downregulation of bFGF-2 in the ventral retina correlated with the increased b-wave amplitude at 45 days, as reported in Valter et al. [40]. More studies will be necessary to unveil the mechanisms by which worsening of the retinal function occurred after this time point.

Thus, the initial decrease in retinal function might have been due to a combination of OS shortening, the inhibitory effect of bFGF-2, and the change in retinal resistance due to gliosis. In other words, as the decreases in the quantified GFAP, bFGF-2, and Iba1^+^ cells accompanied an increase in OS length, a gradual gain in function occurred. Afterward, in the last stage (at 210 days), severe photoreceptor degeneration seemed to overcome retinal functional restoration. Interestingly, the inner retina appeared disarranged at later stages; synaptophysin expression was reduced, and dendritic arborization of ChAT^+^ cells became disorganized. These results suggest that the damage initially triggered by the bright light exposure was too severe such that the intrinsic protective mechanisms of the retina were insufficient to stop its deterioration. Interestingly, the evaluation of synaptophysin expression was discordant with the results obtained in a mouse model by Luis Montalbán-Soler et al. [64]. This difference could be explained by species-dependent differences or by different mechanisms activated through various light damaging protocols; it must be noted that mice (differences exist among strains) do not present a hot spot.

Nonetheless, it is worth noting that most of the retinal disorganization seen in our model was observed in the dorsal retina for up to 7 months, with a rapid loss of photoreceptors followed by substantial disorganization of the inner retina. Instead, the ventral side showed parallel reductions in the thickness of both the ONL and the inner retina. In this chronic phase (i.e., months after the cessation of light exposure), the degree of retinal damage was proportional to the length of the recovery period. The surviving photoreceptors were responsible for maintaining the a-wave. Future studies should look more profoundly at the synaptic and cellular reorganization in the animals inner retina to better understand the retinal network changes. If the outer retina is affected, the signals sent to the inner retina would also be influenced, and consequently, the response of second-order neurons would be impaired. These modifications must lead to substantial changes in the receptive field properties of retinal ganglion cells that are ultimately reflected in altered visual performance [26,68]. Are these changes occurring only at a later stage of the pathology? Are these permanent changes, or can they be restored after the substitution of defective photoreceptors?

It is clear that the ability to detect these pathologies at an early stage is essential for any attempt at restoring retinal function, reducing the impairment of the inner retina, or increasing the chance of successful response to future therapies. Additionally, our data may provide valuable information for developing retinal prosthetics more efficiently and improving their integration with the patient’s retina. Furthermore, it will be fundamental to adopt a more realistic approach that considers the degeneration in the outer retina and the rewiring of the remaining circuit.

## Figures and Tables

**Table 1:** Experimental groups.

Group	Treatment	Light Exposure Duration (hours)	Days (d) after Light Exposure
**HC**	Healthy Control	-	-
**LD12h7d**	LD 1000 lux	12 (short-term)	7
**LD15h7d**		15	
**LD18h7d**		18	
**LD24h0d**		24 (long-term)	0
**LD24h7d**			7
**LD24h15d**			15
**LD24h30d**			30
**LD24h45d**			45
**LD24h60d**			60
**LD24h90d**			90
**LD24h210d**			210

**Table 2:** Primary and secondary antibodies used on retinal cryosections in this study.

**Primary Antibodies**	**Supplier**	**Host Organism**	**Dilution**	**Product#**	**Ref**
Anti-ionized calcium-binding adaptor molecule 1 (Iba1)	Wako	Rabbit	1:1000	019–19741	[42]
Anti-glial fibrillary acidic protein (GFAP)	Dako	Rabbit	1:500	Z0334	[41]
Anti-choline acetyltransferase (ChAT)	Millipore (Chemicon)	Goat	1:100	AB144P	[43]
Anti-L/M Opsin	Millipore (Chemicon)	Rabbit	1:100	AB5405	[41]
Anti-fibroblast growth factor (FGF)−2/basic FGF	Millipore	Mouse	1:200	05–117	[14]
Anti-synaptophysin (SYN)	Osenses	Rabbit	1:300	OSS00021W	
**Secondary Antibodies**	**Supplier**	**Host Organism**	**Dilution**	**Product#**	
Anti-rabbit IgG (H + L) Alexa Fluor 488	Thermo Fisher Scientific	Goat	1:500	A−11008	
Anti-rabbit IgG (H + L) Alexa Fluor 594	Thermo Fisher Scientific	Goat	1:500	A−11012	
Anti-goat IgG (H + L) biotinylated	Thermo Fisher Scientific	Rabbit	1:300	31732	
Anti-mouse IgG (H + L) Alexa Fluor 488	Thermo Fisher Scientific	Goat	1:500	A−11001	

## Data Availability

The data that support the findings of this study are available on request from the corresponding author, S.D.M.
